# Nonlinear relationship between body fat percentage and NAFLD mediated by METS-IR: threshold effects and subgroup differences

**DOI:** 10.1038/s41598-025-10765-w

**Published:** 2025-07-10

**Authors:** Haiyuan Zhao, Yongxin Fang, Jun Zhao, Nianzhao Yang, Yang Li, Fubao Liu, Xiaopeng Chen

**Affiliations:** 1 The First Affiliated Hospital of Anhui Medical University, Hefei, Anhui China; 2 Department of General Surgery, The First Affiliated Hospital of Wannan Medical College (Yijishan Hospital of Wannan Medical College), Wuhu, Anhui China

**Keywords:** Non-alcoholic fatty liver disease (NAFLD), Body fat percentage (BFR), Metabolic score for insulin resistance (METS-IR), Insulin resistance, Mediation analysis, Threshold effect, NHANES, Diseases, Endocrinology, Risk factors

## Abstract

The mechanism linking body fat percentage (BFR) and non-alcoholic fatty liver disease (NAFLD) remains unclear. This study investigated whether the metabolic score for insulin resistance (METS-IR) mediates this relationship and examined threshold effects and population heterogeneity. In this cross-sectional study of 6281 participants from the 2011–2018 National Health and Nutrition Examination Survey (NHANES), logistic regression with restricted cubic splines was used to evaluate the BFR–NAFLD association and identify a threshold. A causal mediation analysis quantified the proportion of the BFR–NAFLD association mediated by METS-IR, and subgroup analyses examined differences by sex, race, and income. BFR was positively associated with NAFLD (OR per 1% increase = 1.12, 95% CI 1.06–1.20), with a threshold at 25.103% BFR; below this point, NAFLD risk rose sharply (OR = 1.61) and above it the association plateaued (OR = 1.09). METS-IR mediated 84.7% (95% CI 80.5–90%) of the BFR–NAFLD association and exhibited an inflection at 36.066 (OR = 1.72 below vs. 1.47 above this value). The BFR–NAFLD association was stronger in men (OR = 1.32), non-Hispanic Blacks (OR = 1.15), and low-income individuals (OR = 1.13) (P for interaction < 0.05). METS-IR mediates most of the relationship between body fat and NAFLD, with a clear threshold effect observed. Early intervention targeting insulin resistance may curb NAFLD progression, especially in individuals with BFR below 25%. These findings provide epidemiological evidence for the metabolic mechanism of NAFLD and inform precision prevention strategies.

**Supplementary Information:**

The online version contains supplementary material available at 10.1038/s41598-025-10765-w.

## Introduction

Metabolic dysfunction-associated steatotic liver disease (MASLD), formerly known as non-alcoholic fatty liver disease (NAFLD), is the most prevalent chronic liver disorder worldwide, with its incidence steadily rising in parallel with the global surge of metabolic syndrome^1–3^. MASLD not only heralds hepatic dysfunction but is also closely intertwined with various metabolic conditions such as cardiovascular disease and diabetes, posing a significant public health challenge^4,5^.

NAFLD, in particular, has emerged as one of the leading chronic liver diseases affecting global populations, characterized by excessive hepatic lipid accumulation^6^. Without timely intervention, NAFLD can progress to non-alcoholic steatohepatitis (NASH), and subsequently advance to hepatic fibrosis, cirrhosis, and even hepatocellular carcinoma (HCC), thereby imposing a substantial burden on public health systems worldwide^7,8^. Despite its complex pathophysiology, extensive research has identified hepatic lipid deposition—strongly associated with obesity, insulin resistance, and metabolic dysregulation—as a core feature of NAFLD^9^. Accordingly, elucidating the precise metabolic mechanisms underlying the relationship between obesity and NAFLD is of critical importance for early prevention and the development of personalized interventions.

Historically, body mass index (BMI) has been the standard metric for assessing the association between obesity and fatty liver disease^10,11^. However, BMI merely reflects the ratio of total body weight to height and fails to distinguish between adipose and lean tissue, nor does it accurately capture the differential distribution of fat between visceral and subcutaneous compartments^12,13^. Growing evidence suggests that the metabolic risks associated with adipose tissue are highly dependent on its distribution, with visceral fat accumulation being more strongly linked to insulin resistance, systemic inflammation, and hepatic steatosis^14,15^. Consequently, body fat percentage (BFR), which provides a direct measure of total body adiposity, has garnered increasing attention as a more precise and sensitive indicator of obesity compared to BMI. Nevertheless, epidemiological evidence concerning the relationship between BFR and NAFLD remains limited and inconsistent, and whether this association follows a linear or nonlinear pattern has yet to be fully elucidated.

In recent years, novel metabolic indices have been proposed for the early detection of insulin resistance and associated metabolic disorders. Among them, the metabolic score for insulin resistance (METS-IR) has attracted considerable interest. METS-IR integrates multiple metabolic parameters, including fasting plasma glucose, triglycerides, BMI, and high-density lipoprotein cholesterol (HDL-C), providing a comprehensive assessment of insulin resistance and its related metabolic disturbances^16–18^. Preliminary studies have suggested that METS-IR may outperform conventional single indicators, such as fasting glucose or the homeostatic model assessment for insulin resistance (HOMA-IR), in evaluating fatty liver risk^19–21^. Recent research by Abenavoli et al. further supports the utility of METS-IR in diagnosing MASLD. In their study involving patients with inflammatory bowel disease, METS-IR demonstrated the highest predictive accuracy for MASLD onset among various metabolic scores and lipid ratios, with an area under the curve (AUC) of 0.754. This finding underscores the potential of METS-IR as a reliable non-invasive biomarker for early MASLD detection^22^.However, robust evidence remains lacking regarding whether—and how—METS-IR mediates the relationship between BFR and NAFLD. Furthermore, the potential threshold effect of METS-IR and its heterogeneity across populations warrant in-depth investigation.

To address these gaps, the present study leveraged data from the large, nationally representative U.S. National Health and Nutrition Examination Survey (NHANES) to systematically explore the relationship between BFR and NAFLD, identify potential nonlinear threshold effects, and further elucidate the mediating role of METS-IR in this association. Our findings are expected not only to advance the understanding of the metabolic mechanisms linking obesity and fatty liver disease but also to provide novel scientific evidence and practical guidance for early risk stratification and personalized intervention strategies in NAFLD prevention.

## Results

### Baseline characteristics

Table 1 summarizes the baseline characteristics of the study population. In total, 6 281 adults from the 2011–2018 NHANES cycles were included, of whom 2 019 were classified as having NAFLD. Compared to individuals without NAFLD, those in the NAFLD group exhibited markedly worse metabolic profiles. Specifically, participants with NAFLD had significantly higher BMI (34.50 vs. 24.31 kg/m², *P* < 0.001), elevated TG levels (177.47 vs. 88.11 mg/dL, *P* < 0.001), increased ALT (31.68 vs. 20.31 U/L, *P* < 0.001), higher AST (26.81 vs. 22.83 U/L, *P* < 0.001), and higher GGT levels (37.63 vs. 17.87 U/L, *P* < 0.001), as well as higher fasting glucose (105.46 vs. 91.04 mg/dL, *P* < 0.001) and total cholesterol concentrations (197.75 vs. 179.23 mg/dL, *P* < 0.001). Conversely, serum albumin (ALB) levels were slightly lower in the NAFLD group (4.22 vs. 4.36 g/dL, *P* < 0.001).

**Table 1:** Baseline characteristics and between-group comparisons

Variable	Total (*n* = 6281)	Non-NAFLD (*n* = 4262)	NAFLD (*n* = 2019)	Statistic	*P*
Age, mean (SE)	35.15 (0.23)	32.47 (0.29)	40.22 (0.38)	t = 16.23	< 0.001
Sex, n (%)				χ^2^ = 46.01	< 0.001
Male	3131 (50.42)	2041 (47.31)	1090 (56.31)		
Female	3150 (49.58)	2221 (52.69)	929 (43.69)		
Race, n (%)				χ^2^ = 62.92	< 0.001
Mexican American	1058 (11.47)	646 (10.17)	412 (13.93)		
Other Hispanic	646 (7.40)	421 (7.23)	225 (7.72)		
Non-Hispanic White	2096 (60.83)	1378 (60.55)	718 (61.36)		
Non-Hispanic Black	1327 (10.89)	888 (10.77)	439 (11.13)		
Other race	1154 (9.40)	929 (11.28)	225 (5.85)		
Education level custom, n (%)				χ^2^ = 38.43	< 0.001
Below high school	288 (4.12)	143 (3.47)	145 (5.10)		
High school	1532 (31.21)	843 (28.92)	689 (34.68)		
Above high school	2747 (64.67)	1748 (67.61)	999 (60.22)		
PIR custom, n (%)				χ^2^ = 26.89	< 0.001
< 1.0	1461 (17.57)	980 (17.03)	481 (18.58)		
1.0–3.0	2360 (36.69)	1551 (34.87)	809 (40.09)		
> 3.0	1944 (45.73)	1381 (48.10)	563 (41.32)		
Hypertension, n (%)				χ^2^ = 474.99	< 0.001
No	4116 (75.94)	2942 (84.99)	1174 (60.67)		
Yes	1278 (24.06)	510 (15.01)	768 (39.33)		
Diabetes, n (%)				χ^2^ = 400.48	< 0.001
No	5644 (91.86)	4065 (96.86)	1579 (82.33)		
Yes	574 (8.14)	161 (3.14)	413 (17.67)		
BFR, mean (SE)	32.30 (0.18)	29.88 (0.21)	36.87 (0.23)	t = 20.72	< 0.001
METS IR, mean (SE)	40.99 (0.29)	34.17 (0.14)	53.87 (0.32)	t = 59.34	< 0.001
WT, mean (SE)	79.29 (0.42)	68.73 (0.24)	99.26 (0.57)	t = 53.54	< 0.001
HT, mean (SE)	168.47 (0.16)	167.81 (0.21)	169.70 (0.26)	t = 5.80	< 0.001
BMI, mean (SE)	27.83 (0.15)	24.31 (0.08)	34.50 (0.20)	t = 48.11	< 0.001
ALB, mean (SE)	4.31 (0.01)	4.36 (0.01)	4.22 (0.01)	t = − 10.65	< 0.001
ALT, mean (SE)	24.25 (0.30)	20.31 (0.32)	31.68 (0.59)	t = 16.61	< 0.001
AST, mean (SE)	24.20 (0.24)	22.83 (0.27)	26.81 (0.51)	t = 6.82	< 0.001
BUN, mean (SE)	12.43 (0.08)	12.31 (0.11)	12.65 (0.12)	t = 2.10	0.040
TC, mean (SE)	185.64 (0.70)	179.23 (0.82)	197.75 (1.31)	t = 12.46	< 0.001
GGT, mean (SE)	24.71 (0.45)	17.87 (0.41)	37.63 (0.95)	t = 20.63	< 0.001
GLU, mean (SE)	96.03 (0.44)	91.04 (0.41)	105.46 (0.82)	t = 16.28	< 0.001
LDH, mean (SE)	130.06 (0.59)	127.97 (0.70)	134.00 (1.02)	t = 4.86	< 0.001
TBIL, mean (SE)	0.64 (0.01)	0.66 (0.01)	0.60 (0.01)	t = − 5.02	< 0.001
TP, mean (SE)	7.14 (0.01)	7.14 (0.01)	7.14 (0.02)	t = − 0.36	0.719
TG, mean (SE)	119.02 (2.16)	88.11 (1.06)	177.47 (5.01)	t = 17.71	< 0.001
UA, mean (SE)	5.30 (0.03)	4.98 (0.03)	5.91 (0.03)	t = 23.67	< 0.001
Cr, mean (SE)	0.82 (0.00)	0.81 (0.01)	0.84 (0.01)	t = 4.22	< 0.001
GLOB, mean (SE)	2.83 (0.01)	2.78 (0.01)	2.92 (0.01)	t = 10.80	< 0.001
WBC, mean (SE)	6.76 (0.04)	6.40 (0.04)	7.43 (0.06)	t = 16.06	< 0.001
LYMPH, mean (SE)	2.09 (0.01)	2.03 (0.01)	2.21 (0.02)	t = 9.00	< 0.001
NEUT, mean (SE)	3.89 (0.03)	3.63 (0.03)	4.39 (0.04)	t = 13.70	< 0.001
RBC, mean (SE)	4.79 (0.01)	4.74 (0.01)	4.90 (0.01)	t = 10.56	< 0.001
PLT, mean (SE)	239.71 (1.21)	235.21 (1.22)	248.23 (1.94)	t = 6.32	< 0.001

*SE* standard error, *t* t-test, *χ²* Chi-square test, *BFR* body fat rate, *CI* confidence interval, *NAFLD* non-alcoholic fatty liver disease, *OR* odds ratio

Moreover, the prevalence of hypertension (39.3% vs. 15.0%) and diabetes (17.7% vs. 3.1%) was significantly higher among participants with NAFLD compared to those without (*both P* < 0.001). Demographically, NAFLD was more common in men and in individuals from lower-income households. Within the NAFLD cohort, 61.4% were non-Hispanic White, 13.9% Mexican American, 11.1% non-Hispanic Black, 7.7% other Hispanic, and 5.9% another race (χ² = 62.92, *P* < 0.001). Significant differences were observed across sex, race/ethnicity, and poverty-income ratio categories (all *P* < 0.001).

### Multivariable logistic regression analysis of BFR and NAFLD risk

Table 2 summarises the multivariable logistic-regression results for BFR and NAFLD risk .Across all multivariable logistic regression models, higher BFR was consistently associated with an increased risk of NAFLD. In the unadjusted model (Model 1), each 1% increment in BFR was associated with an 11% increase in NAFLD risk (OR = 1.11, 95% CI 1.10–1.12, *P* < 0.001). After adjusting for sex, race/ethnicity, education, age, hypertension, diabetes, and income (Model 2), the association remained significant (OR = 1.09, 95% CI 1.05–1.13; *P* < 0.001).Further adjustment for all biochemical and anthropometric variables (Model 3) yielded consistent results, with BFR still showing a robust association with NAFLD risk (OR = 1.12, 95% CI 1.06–1.20, *P* < 0.001).

**Table 2:** Multivariable logistic regression analysis of BFR and NAFLD risk

Variables	Model1		Model2		Model3	
	OR (95%CI)	*P*	OR (95%CI)	*P*	OR (95%CI)	*P*
BFR continuous	1.11 (1.10–1.12)	< 0.001	1.09 (1.05–1.13)	< 0.001	1.12 (1.06–1.20)	< 0.001
BFR quantile						
Q1	1.00		1.00		1.00	
Q2	5.37 (4.10–7.02)	< 0.001	2.57 (1.55–4.25)	< 0.001	4.42 (2.06–9.47)	< 0.001
Q3	4.99 (3.71–6.72)	< 0.001	3.28 (1.76–6.12)	< 0.001	5.83 (2.26–15.04)	0.001
Q4	14.14 (10.75–18.60)	< 0.001	3.66 (1.80–7.44)	< 0.001	5.29 (1.67–16.70)	0.009
P for trend		< 0.001		< 0.001		< 0.001

*OR* odds ratio, *CI* confidence interval

Model1: Crude

Model2: Adjust: Adjusted for sex, race/ethnicity, education level, hypertension, diabetes, age, and PIR

Model3: Adjust: Adjusted for sex, race/ethnicity, education level, hypertension, diabetes, albumin (ALB), alanine aminotransferase (ALT), aspartate aminotransferase (AST), blood urea nitrogen (BUN), total cholesterol (TC), gamma-glutamyl transferase (GGT), fasting glucose (GLU), lactate dehydrogenase (LDH), total bilirubin (TBIL), total protein (TP), triglycerides (TG), uric acid (UA), creatinine (Cr), globulin (GLOB), white blood cell count (WBC), lymphocyte count (LYMPH), neutrophil count (NEUT), red blood cell count (RBC), platelet count (PLT), weight (WT), height (HT), BMI, age, and PIR

Quartile-based analyses further confirmed a stepwise increase in NAFLD risk with higher BFR levels. In the unadjusted model, compared to the lowest BFR quartile (Q1), the odds of NAFLD were markedly higher in Q2 (OR = 5.37, 95% CI 4.10–7.02), Q3 (OR = 4.99, 95% CI 3.71–6.72), and Q4 (OR = 14.14, 95% CI 10.75–18.60), all with *P* < 0.001. After adjustment in Model 2, this upward trend persisted (Q2: OR = 2.57; Q3: OR = 3.28; Q4: OR = 3.66; all *P* < 0.001). Even in the fully adjusted Model 3, elevated risks were consistently observed across higher BFR quartiles: Q2 (OR = 4.42, 95% CI 2.06–9.47, *P* < 0.001), Q3 (OR = 5.83, 95% CI 2.26–15.04, *P* = 0.001), and Q4 (OR = 5.29, 95% CI 1.67–16.70, *P* = 0.009). A statistically significant linear trend was observed across all models (*P* for trend < 0.001).

**Fig. 1:**
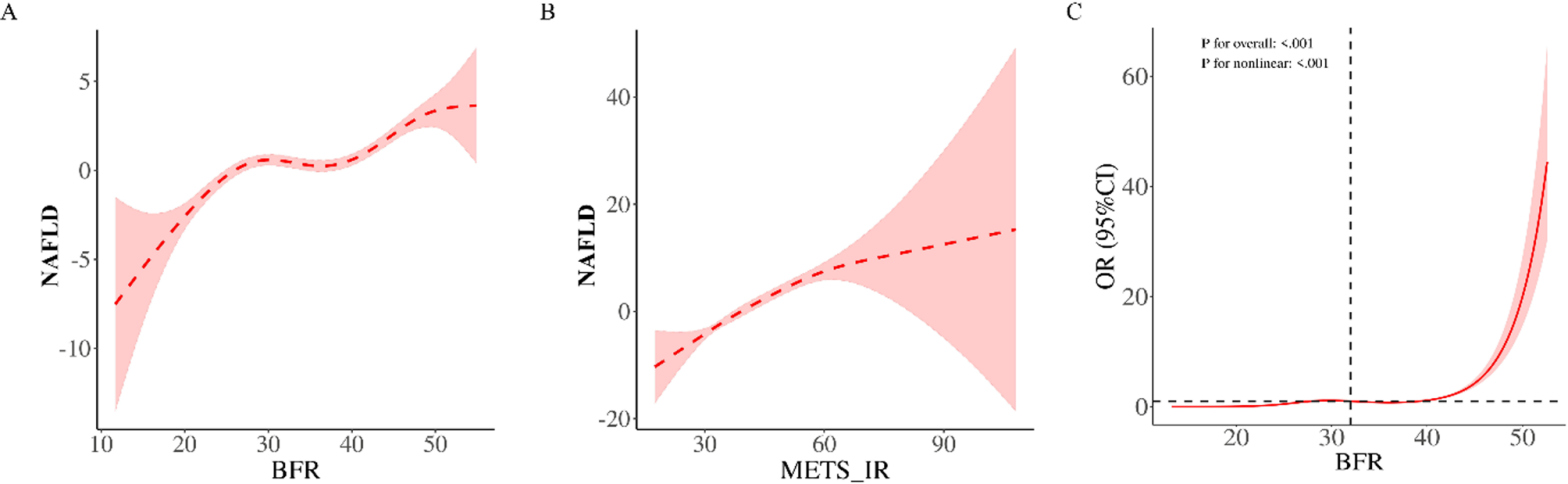
The flowchart of this study

### Nonlinear associations and threshold effects (RCS analysis)

**Fig. 2:**
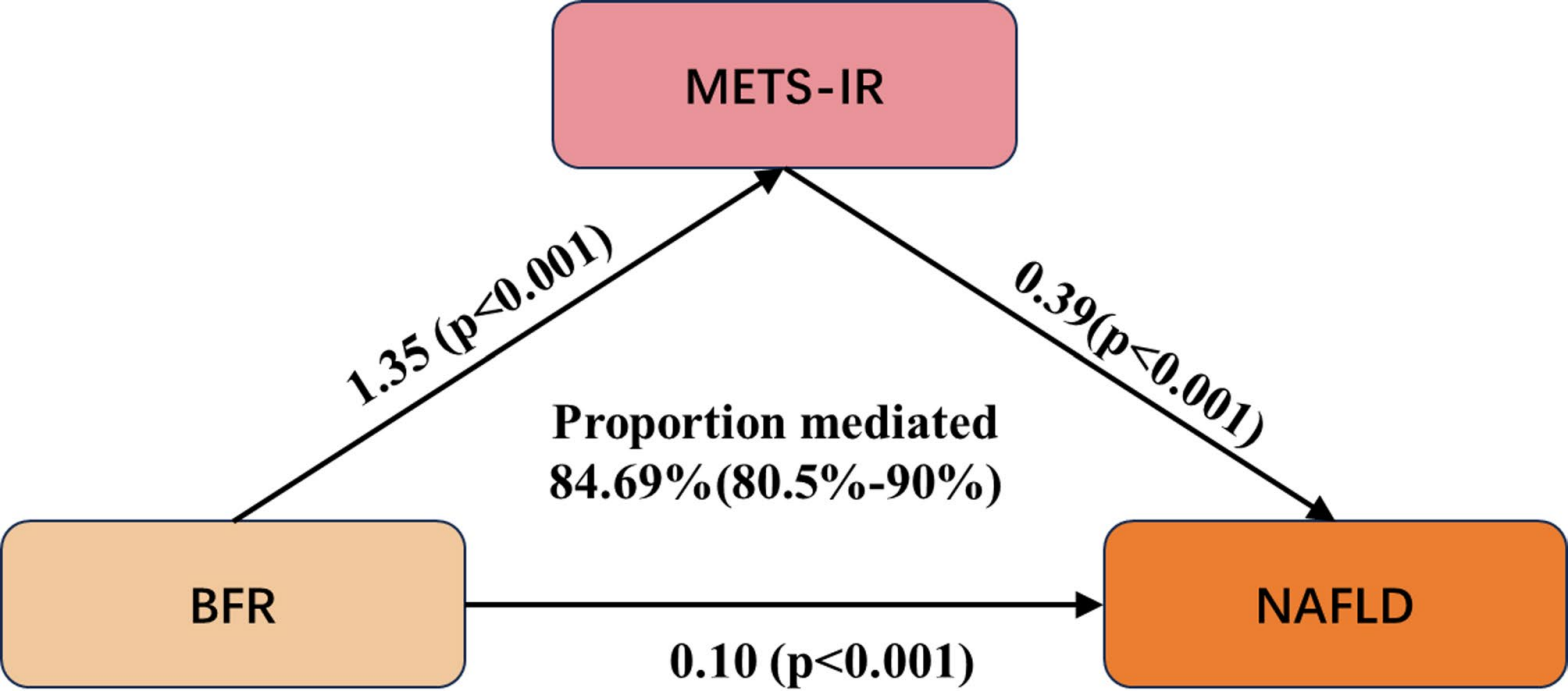
Nonlinear associations of BFR and METS-IR with NAFLD risk. **A** Dose–response curve depicting the relationship between BFR and NAFLD risk. The solid line represents the odds ratio (OR), and the shaded area indicates the 95% confidence interval. The inflection point is identified at BFR = 25.103%; below this threshold, the risk increases sharply (OR = 1.61), while above it, the risk continues to rise but at a reduced slope (OR = 1.09). **B** Dose–response curve illustrating the association between METS-IR and NAFLD risk. The threshold is observed at METS-IR = 36.066; below this value, the risk escalates rapidly (OR = 1.72), whereas above it, the risk remains elevated but increases at a slower rate (OR = 1.47). **C** Restricted cubic spline curve demonstrating the association between BFR and NAFLD risk, using BFR = 32% as the reference point. The model incorporates knots at 17.9%, 28.3%, 36.1%, and 46.2% to capture the nonlinear association

Figure 2A and Supplementary Table 1 present the *threshold* (segmented-regression) analysis for body-fat percentage (BFR). A data-driven breakpoint was detected at 25.103% BFR. Below this point, each 1% rise in BFR raised NAFLD risk by **61%** (OR = 1.61, 95% CI 1.43–1.83; *P* < 0.001), whereas above 25.103% the increment declined to **9%** (OR = 1.09, 95% CI 1.08–1.10; *P* < 0.001). A subsequent restricted-cubic-spline (RCS) curve—reference 32% and knots at 17.9%, 28.3%, 36.1%, 46.2%—confirmed a highly significant non-linear association (*P* for non-linearity < 0.001) and graphically illustrated the plateau beyond the breakpoint.

Figure 2B and Supplementary Table 2 display the analogous threshold analysis for METS-IR. The optimal cut-off was 36.066; each one-unit increase below this value raised NAFLD risk by **72%** (OR = 1.72, 95% CI 1.23–2.42; *P* = 0.002), while above 36.066 the increment fell to **47%** (OR = 1.47, 95% CI 1.43–1.51; *P* < 0.001). The corresponding RCS model again demonstrated strong non-linearity (*P* < 0.001), underscoring the cumulative impact of metabolic dysfunction on NAFLD risk.

Figure 2C overlays the full RCS curves for both predictors, using 32% BFR as the reference and the same four knots (17.9%, 28.3%, 36.1%, 46.2%) to depict the complete, non-linear dose–response patterns.

### Mediation analysis of METS-IR

A series of regression analyses were conducted to elucidate how the exposure variable (BFR) influences the mediator (METS-IR) and, in turn, the outcome variable (NAFLD).First, the regression of exposure on the mediator demonstrated a significant positive association between BFR and METS-IR (β = 1.32, S.E. = 0.03, *t* = 49.68, *P* < 0.001), indicating that each one-unit increase in BFR corresponded to an increase of 1.32 units in METS-IR (95% CI 1.27–1.37).

Second, regression analysis between the exposure and the outcome revealed that BFR was also significantly and positively associated with NAFLD (β = 0.31, S.E. = 0.01, *t* = 27.92, *P* < 0.001), corresponding to an OR of 1.37 (95% CI 1.34–1.40). This indicates that each unit increase in BFR was associated with a 37% higher likelihood of developing NAFLD (95% CI 34–40%).

Further regression analysis incorporating both the exposure and the mediator confirmed the persistent significant effect of BFR on METS-IR (β = 1.32, S.E. = 0.03, *t* = 49.68, *P* < 0.001), while also demonstrating a significant positive effect of METS-IR on FLI_custom (β = 0.41, S.E. = 0.02, *t* = 20.14, *P* < 0.001), with an OR of 1.51 (95% CI 1.45–1.58). This suggests that each unit increase in METS-IR was associated with a 51% greater likelihood of NAFLD occurrence (95% CI 45–58%).

**Fig. 3:**
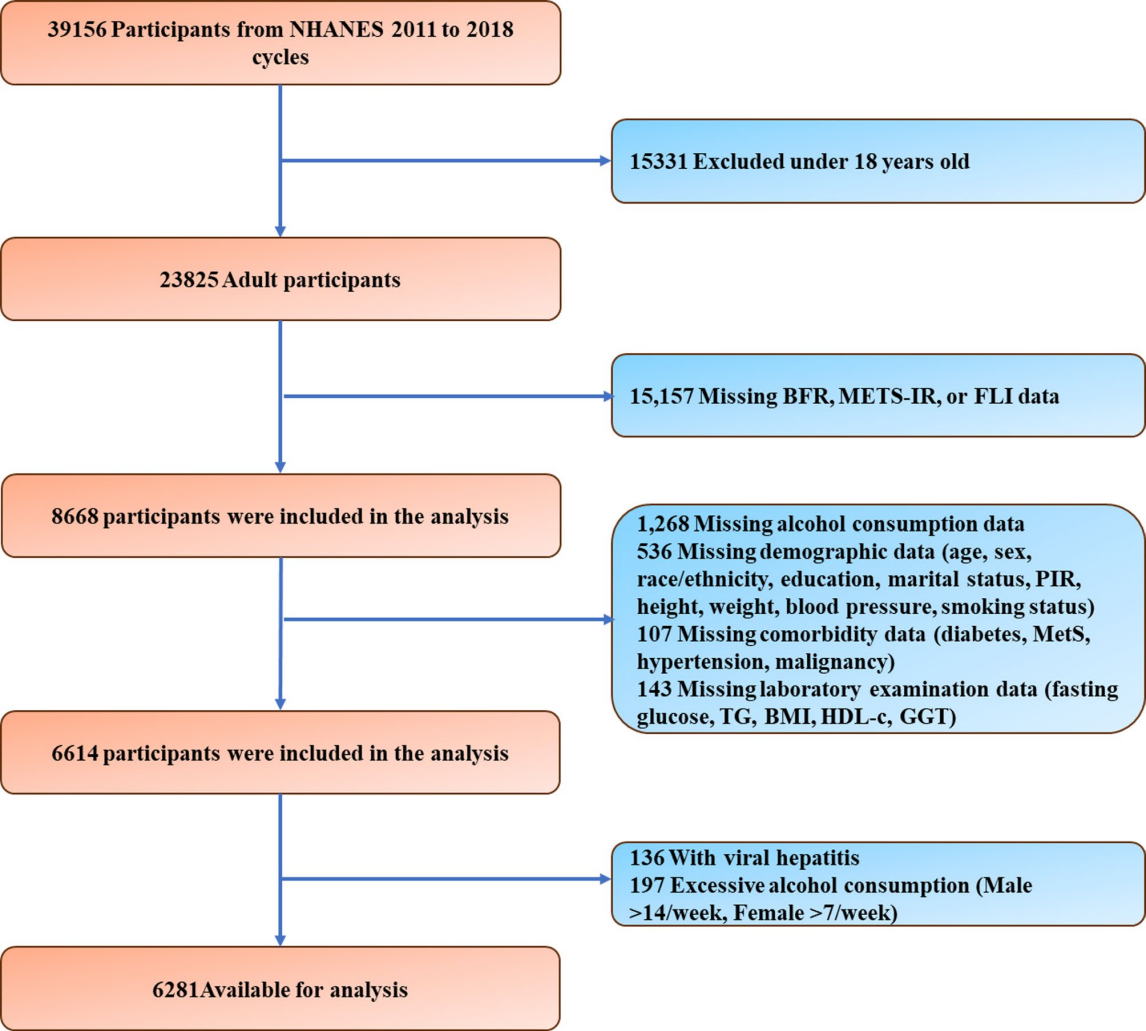
Mediation model of METS-IR in the pathway from BFR to NAFLD. The path diagram illustrates both the direct and indirect effects of BFR on NAFLD mediated through METS-IR. Solid arrows represent the estimated effect sizes

Mediation analysis further revealed that the indirect effect accounted for 82.84% of the total effect of BFR on the outcome, while the direct effect accounted for 17.16%, indicating that METS-IR plays a substantial mediating role in the relationship between BFR and NAFLD. Collectively, these findings suggest that BFR increases the risk of NAFLD primarily through its impact on elevating METS-IR levels, and this mediating pathway was statistically significant (see Fig. 3 and Supplementary Table 3.x).

### Subgroup analysis: impact of body fat percentage on NAFLD risk

Table 3 summarises the stratified analyses conducted to examine how sex, race/ethnicity, household income (PIR), education, and metabolic-syndrome status modify the association between BFR and NAFLD. To further assess the factors influencing NAFLD and their associations across various subgroups, we conducted a series of stratified analyses. These analyses explored the effects of sex, race/ethnicity, household income level (PIR), educational background, and metabolic syndrome status. In the overall population, the OR was 1.11 (95% CI 1.10–1.12, *P* < 0.001), indicating a significant association between BFR and NAFLD risk across the sample .

**Table 3:** Subgroup analysis of the impact of sex, race/ethnicity, and income level on NAFLD risk

Variables	*n* (%)	OR (95% CI)	*P*	*P* for interaction
All patients	6281 (100.00)	1.11 (1.10–1.12)	< 0.001	
Sex				< 0.001
Male	3131 (49.85)	1.32 (1.29–1.35)	< 0.001	
Female	3150 (50.15)	1.45 (1.40–1.49)	< 0.001	
Race				0.032
Mexican American	1058 (16.84)	1.09 (1.07–1.11)	< 0.001	
Other Hispanic	646 (10.28)	1.10 (1.08–1.13)	< 0.001	
Non- Hispanic White	2096 (33.37)	1.11 (1.10–1.13)	< 0.001	
Non-Hispanic Black	1327 (21.13)	1.15 (1.13–1.16)	< 0.001	
Other race	1154 (18.37)	1.11 (1.07–1.14)	< 0.001	
PIR n (%)				0.020
< 1.0	1461 (25.34)	1.13 (1.11–1.15)	< 0.001	
1.0–3.0	2360 (40.94)	1.12 (1.10–1.14)	< 0.001	
> 3.0	1944 (33.72)	1.09 (1.08–1.11)	< 0.001	
Education level n (%)				0.425
Below high school	288 (6.31)	1.09 (1.06–1.13)	< 0.001	
High school	1532 (33.54)	1.10 (1.08–1.12)	< 0.001	
Above high school	2747 (60.15)	1.11 (1.10–1.13)	< 0.001	
METS_IR				0.124
≤ 39.12	3306 (52.63)	1.11 (1.05–1.17)	< 0.001	
> 39.12	2975 (47.37)	1.06 (1.05–1.07)	< 0.001	

*OR* odds ratio, *CI* confidence interval

Specifically, the OR for men was 1.32 (95% CI 1.29–1.35, *P* < 0.001), while for women it was 1.45 (95% CI 1.40–1.49, *P* < 0.001), suggesting a notable sex-specific difference in the association with NAFLD. Regarding race/ethnicity, non-Hispanic Black participants exhibited an OR of 1.15 (95% CI 1.13–1.16, *P* < 0.001), which was higher than that observed in other groups such as Mexican Americans (OR = 1.09, 95% CI 1.07–1.11) and other Hispanic populations (OR = 1.10, 95% CI 1.08–1.13).

Income-stratified analysis revealed that individuals in the low-income category (PIR < 1.0) had an OR of 1.13 (95% CI 1.11–1.15, *P* < 0.001), those in the middle-income group (PIR 1.0–3.0) had an OR of 1.12 (95% CI 1.10–1.14, *P* < 0.001), and participants in the high-income group (PIR > 3.0) showed an OR of 1.09 (95% CI 1.08–1.11, *P* < 0.001).

With respect to educational background, although significant associations were observed across all levels of education, the differences between groups were relatively minor (*P* = 0.425). When stratified by metabolic syndrome status, participants with a METS-IR median value ≤ 39.12 had an OR of 1.11 (95% CI 1.05–1.17, *P* < 0.001), while those with METS-IR > 39.12 had an OR of 1.06 (95% CI 1.05–1.07, *P* < 0.001), with no statistically significant interaction effect observed (*P* = 0.124).

In summary, In summary, sex, race/ethnicity, and socioeconomic status exert the strongest modifying effects on the BFR–NAFLD association, whereas educational attainment and metabolic-syndrome status show comparatively weaker interactions.

## Discussion

Drawing upon data from the NHANES, this study systematically elucidated the nonlinear relationship between BFR and NAFLD risk and, for the first time, quantitatively characterized the pivotal mediating role of METS-IR in this association. Our findings demonstrated a significant positive correlation between BFR and NAFLD risk, with a clear threshold effect identified at BFR = 25.103%. Below this threshold, each unit increase in BFR was associated with a sharp elevation in NAFLD risk (OR = 1.61), while above this threshold, the magnitude of risk increase was notably attenuated (OR = 1.09). This “threshold effect” may reflect a stage-specific shift in fat distribution and metabolic consequences. At lower body fat levels, incremental fat tends to accumulate preferentially as visceral adipose tissue, which is metabolically active and readily releases free fatty acids (FFAs) into the portal circulation, thereby substantially promoting hepatic lipid deposition^23^. Matsuzawa et al. previously reported that visceral fat is metabolically more active than subcutaneous fat, mobilizing large amounts of FFAs to the liver and leading to steatosis. Therefore, even modest increases in visceral fat at lower BFR levels can markedly exacerbate insulin resistance and hepatic fat accumulation, sharply elevating NAFLD risk^24^. Conversely, as total body fat increases, further accumulation predominantly occurs in subcutaneous depots, which serve as a relatively “inert” buffer for excess FFAs, thereby mitigating hepatic lipotoxicity. Changes in the visceral-to-subcutaneous fat ratio may thus explain the observed plateauing of NAFLD risk beyond the BFR threshold^25^. Furthermore, at very high BFR levels, it is plausible that most susceptible individuals have already developed NAFLD, leaving a residual population with protective factors that diminish the marginal impact of further BFR increases^26^. These potential mechanisms are consistent with our observed nonlinear patterns^27,28^. Collectively, these findings underscore the need for heightened vigilance in individuals with relatively low overall body fat but a propensity for visceral fat accumulation, as prevention of further fat gain at this stage may be critical for reducing NAFLD risk.

Building upon this, Insulin resistance emerged as a central mediator in this association. Mediation analysis quantified that METS-IR accounted for approximately 84.7% of the effect of BFR on NAFLD risk, indicating that the majority of the impact of increased BFR on NAFLD is mediated through insulin resistance. This aligns with existing evidence recognizing obesity-induced insulin resistance as a key driver of NAFLD pathogenesis^29^. Numerous studies have demonstrated that NAFLD prevalence is markedly higher in insulin-resistant states, including obesity, type 2 diabetes, dyslipidemia, and metabolic syndrome^30,31^. Our findings reinforce this understanding by providing quantitative support. Mechanistically, insulin resistance promotes hepatic uptake of FFAs, impairs hepatic insulin signaling, and disrupts lipid metabolism, thereby accelerating hepatic fat accumulation^32^. These processes help explain the high propensity for NAFLD development in individuals with elevated body fat. A novel contribution of our study is the elucidation and precise quantification of the mediating role of METS-IR in the BFR–NAFLD pathway, offering direct evidence for the “obesity–insulin resistance–NAFLD” pathogenic axis. Notably, we also observed a threshold effect in the METS-IR–NAFLD association (with an inflection point around 36), suggesting that beyond a certain level of insulin resistance, the marginal effect on hepatic fat accumulation diminishes. This implies the existence of a physiological “saturation point,” where excessive insulin resistance no longer substantially drives further steatosis, and the disease trajectory may shift toward inflammation and fibrosis^33^. Overall, these results reinforce the central role of insulin resistance in NAFLD development and highlight the potential of early insulin sensitization to significantly mitigate the hepatometabolic risks associated with increased body fat.

Furthermore, our study extends the current understanding in several important ways. First, we demonstrated that BFR, as a direct indicator of total fat mass and distribution, offers distinct advantages over traditional metrics such as BMI. Most existing studies have focused on the linear relationship between BMI and NAFLD, often overlooking the heterogeneity of fat composition^34^. By contrast, our focus on BFR revealed a significant nonlinear threshold effect, a feature not previously well-characterized. Our findings are consistent with those of Ito et al.^35^who, in a Japanese cohort, found that elevated BFR independently predicted hepatic steatosis even after adjusting for BMI, highlighting that individuals with high BMI do not necessarily have high body fat percentages. Indeed, many NAFLD patients are not obese by BMI criteria. Reports estimate that approximately 10–20% of global NAFLD cases occur in “lean NAFLD” populations, characterized by normal BMI but elevated BFR. These individuals often exhibit visceral fat accumulation and subtle metabolic disturbances, placing them at risk for NAFLD despite an ostensibly healthy body weight^36^. This evidence corroborates our findings, emphasizing the limitations of BMI in assessing individual hepatic fat risk and the superior sensitivity of direct BFR measurements. Notably, a large-scale study in an Iranian population identified total body fat percentage as the best predictor of NAFLD, with AUC values of 0.93 for men and 0.92 for women, and identified critical BFR thresholds of approximately 26.7% for men and 32.2% for women^37^. These findings support the rationale for using BFR in NAFLD risk stratification. By incorporating nonlinear modeling, our study proposed an overall threshold of approximately 25%, closely aligning with sex-specific thresholds reported in prior research. This nonlinear characterization offers valuable insights for BFR-based risk stratification of NAFLD, with important clinical implications. BFR, typically assessed via DXA, provides precise measurements of body composition but may be limited by cost and accessibility in routine clinical settings. Bioelectrical impedance analysis (BIA) offers a more affordable and non-invasive alternative; however, its accuracy varies across populations and requires further validation. Our DXA-based study serves as a reference standard for future BIA calibration. METS-IR, calculated from routine laboratory parameters, is easily implementable and has demonstrated consistent predictive performance for NAFLD across diverse populations. Incorporating BFR and METS-IR into routine assessments can facilitate early identification of individuals at high risk for NAFLD. Elevated BFR indicates increased visceral adiposity, while higher METS-IR scores reflect insulin resistance—both associated with greater NAFLD risk. Early detection through these biomarkers enables timely lifestyle modifications and therapeutic interventions, potentially halting or reversing disease progression and improving long-term patient outcomes.

Finally, our study highlights the unique value of METS-IR in NAFLD risk assessment. Compared with traditional indices such as HOMA-IR or fasting insulin, or isolated metabolic markers, METS-IR integrates multiple metabolic dimensions—glucose levels, lipid profile, and adiposity—to comprehensively reflect insulin resistance status^38^. Large population-based studies across diverse cohorts, including non-obese adults, the elderly, and Asian populations, have demonstrated the superior predictive performance of METS-IR for NAFLD risk^21,39^. Our study innovatively incorporated METS-IR into a mediation analysis framework and, for the first time, quantified its contribution to the BFR–NAFLD pathway at 84.7%, confirming its substantial explanatory power for fat accumulation–related liver risk. These findings not only deepen our understanding of METS-IR’s pathophysiological significance but also support its potential application as an early warning tool for NAFLD, with implications for optimizing risk stratification and screening strategies.

Firstly, to our knowledge this is the first study to perform a quantitative mediation analysis to explore the intermediary role of insulin resistance (assessed via the METS-IR index) in the relationship between body fat percentage and NAFLD. Secondly, we identified a non-linear, threshold relationship between BFR and NAFLD risk, indicating a specific body fat percentage beyond which NAFLD risk rises substantially. Additionally, our analysis is based on a large, nationally representative sample, which enhances the generalizability of the findings. Finally, we conducted multiple sensitivity analyses that consistently affirmed our results, thereby strengthening confidence in our conclusions.

However, This study has several limitations. First, the cross-sectional design precludes causal inference regarding the relationship between BFR and NAFLD, necessitating confirmation through prospective cohort studies. Second, while the FLI is a validated tool for epidemiological use, it has inherent limitations in detecting hepatic fibrosis and mild steatosis, and future studies should integrate imaging modalities such as ultrasound, MRI, or liver biopsy for greater diagnostic accuracy. Third, residual confounding factors—including dietary patterns, physical activity, sleep behaviors, gut microbiota, and environmental exposures—may have influenced our results; future models should incorporate more comprehensive lifestyle and genetic data. Fourth, methodological enhancements such as propensity score matching could improve group comparability, and structural equation modeling could better elucidate the complex pathways linking BFR, insulin resistance, and NAFLD, thereby strengthening the robustness of our conclusions. These limitations point toward valuable directions for future research.

Building upon our findings, future research should pursue prospective, multicenter cohort studies to dynamically validate the causal relationship between BFR and NAFLD, particularly to test the temporal relevance of the identified ~ 25% BFR threshold. Further investigations are warranted to explore the interactions between METS-IR and other metabolic biomarkers (e.g., fasting insulin, FFAs, leptin, adiponectin) to clarify the mechanistic pathways of insulin resistance in NAFLD pathogenesis. Designing precision prevention strategies for at-risk subpopulations—especially those with normal BMI but elevated METS-IR—could provide insights into the effectiveness of lifestyle interventions and insulin-sensitizing therapies. Moreover, given our findings that BFR–NAFLD associations were more pronounced among men, non-Hispanic Blacks, and individuals from lower-income groups, targeted screening and intervention strategies tailored to these high-risk subgroups, taking into account cultural and socioeconomic factors, could enhance both the efficacy and equity of prevention efforts.

## Conclusion

This study identified a significant nonlinear threshold relationship between BFR and NAFLD and, for the first time, quantitatively delineated the major mediating role of insulin resistance, as captured by METS-IR, in this association. These findings offer new perspectives for early identification and prevention of NAFLD: compared to traditional BMI-based assessments, monitoring BFR and METS-IR may enable more accurate risk stratification and the development of individualized preventive strategies. Nonetheless, given the study’s limitations, these conclusions warrant further validation in future research. Such efforts will contribute to a deeper understanding of the metabolic mechanisms underlying NAFLD and support the advancement of precision prevention and management strategies.

## Methods

### Study design and data source

This study adopted a cross-sectional design, utilizing publicly available data from the National Health and Nutrition Examination Survey (NHANES) spanning 2011 to 2018. Administered by the National Center for Health Statistics (NCHS) under the Centers for Disease Control and Prevention (CDC), NHANES employs a complex, multistage, stratified, and clustered probability sampling strategy to obtain nationally representative health and nutritional data from the non-institutionalized U.S. population^40^. Each survey cycle spans two years, with deliberate oversampling of specific subpopulations (such as ethnic minorities and the elderly) to enhance the precision and representativeness of estimates. All participants provided written informed consent, and the survey protocol was approved by the NCHS Institutional Review Board, adhering to ethical research standards. Data collection included household interviews, physical examinations, and laboratory tests, with detailed procedures publicly documented by NCHS. Since NHANES data are fully anonymized and freely accessible, the present study was conducted as a secondary data analysis, exempt from additional ethical review. The original datasets and technical documentation for the 2011–2018 survey cycles are available from the NCHS website, ensuring full transparency and reproducibility of the research process.

NHANES sampling weights were rigorously applied to account for the complex survey design. For the four consecutive two-year cycles from 2011 to 2018, individual sample weights were recalibrated in accordance with NCHS analytical guidelines, adjusting the original two-year weights to represent the full eight-year survey period. Furthermore, stratification variables and primary sampling units (PSUs) provided by NHANES were incorporated into our models to appropriately adjust for design effects inherent to the complex sampling framework.

### Study population

The study population was drawn from the cumulative NHANES dataset for 2011–2018, initially comprising 39,156 participants. Predefined inclusion and exclusion criteria were established in accordance with the study objectives, as illustrated in the sample-selection flowchart (Fig. 1). Inclusion criteria were as follows: adults aged 18 years or older with available data on key variables, including BFR, METS-IR^41,42^and the fatty liver index (FLI)^43,44^. Exclusion criteria were applied systematically. Participants were removed if they met any of the following conditions: (1) Age < 18 years (*n* = 15 331; 39.1% of the initial sample), (2) Missing critical variables—BFR, METS-IR, or FLI (*n* = 15 157; 38.7%),(3) Viral hepatitis (diagnosed or self-reported) (*n* = 136; 0.3%),(4) No alcohol-consumption data (*n* = 1 268; 3.2%),(5) Incomplete demographics or anthropometrics (e.g., age, sex, race/ethnicity, education, marital status, PIR, height, weight, blood pressure) (*n* = 536; 1.4%),(6) Incomplete medical history for key conditions (diabetes, metabolic syndrome, hypertension, malignancy) (*n* = 107; 0.3%),(7) Missing essential laboratory measures (e.g., fasting glucose, triglycerides, HDL-C, γ-glutamyl transferase) (*n* = 143; 0.4%),(8) Excessive alcohol intake (*n* = 197; 0.5%),After these exclusions, 6 281 eligible adults remained for analysis.

Excessive alcohol intake was defined as consumption exceeding 14 standard drinks per week for men or 7 drinks per week for women, in order to exclude potential cases of alcoholic fatty liver disease.

Following these sequential exclusions, a final sample of 6,281 eligible adults was retained for analysis. To minimize potential bias from missing data, a complete-case analysis was performed, retaining only participants with complete values for all required variables.

### Variable definitions and measurements

*Body fat percentage (BFR)* The primary exposure variable, BFR, was assessed using dual-energy X-ray absorptiometry (DXA). All participants underwent standardized whole-body composition scans at NHANES Mobile Examination Centers, conducted by certified technicians trained in DXA protocols. These scans provided precise measurements of total fat mass and lean mass, from which the percentage of body fat was subsequently calculated. To ensure the consistency and accuracy of BFR measurements, NHANES implemented rigorous quality control procedures and calibration standards across survey cycles, employing uniform scanning equipment and protocols throughout the study period.

*Diagnosis of non-alcoholic fatty liver disease (NAFLD)* NAFLD, the primary outcome of this study, was identified using the Fatty Liver Index (FLI). FLI is a validated composite score that integrates anthropometric and biochemical parameters, originally developed by Bedogni et al. and subsequently confirmed in multiple independent studies. The FLI is calculated according to the following formula:

$$\:FLI=\left\{\frac{{\text{e}}^{0.953\text{*}{\text{l}\text{o}\text{g}}_{e}\left(\text{triglycerides}\right)+0.139\text{*}\text{B}\text{M}\text{I}+0.718\text{*}{\text{l}\text{o}\text{g}}_{e}\left(\text{G}\text{G}\text{T}\right)+0.053\text{*}\text{waist\:circumference}-15.745}}{1+{\text{e}}^{0.953\text{*}{\text{l}\text{o}\text{g}}_{e}\left(\text{triglycerides}\right)+0.139\text{*}\text{B}\text{M}\text{I}+0.718\text{*}{\text{l}\text{o}\text{g}}_{e}\left(\text{G}\text{G}\text{T}\right)+0.053\text{*}\text{waist\:circumference}-15.745}}\right\}\text{*}100$$

In the FLI calculation, TG represents fasting triglycerides (mg/dL), BMI is body mass index (kg/m²), GGT refers to gamma-glutamyl transferase (U/L), and waist circumference is measured in centimeters (cm). Using this index, an FLI threshold of ≥ 60 was applied: participants with an FLI score ≥ 60 were classified as having NAFLD, whereas those scoring < 60 were considered free of NAFLD. This cutoff has been widely adopted in epidemiological research and demonstrates high specificity for identifying individuals with significant hepatic steatosis. It is important to note that, although FLI is not a direct clinical diagnostic tool, previous studies have shown that an FLI ≥ 60 corresponds to approximately 86% specificity for ultrasonography-confirmed hepatic steatosis, making it a reliable surrogate marker for large-scale population screening^45,46^.

*METS-IR as a potential mediator*^47^

$$\:\text{M}\text{E}\text{T}\text{S}\text{-}\text{I}\text{R}=\frac{\text{l}\text{n}\left(2\times\:\text{F}\text{P}\text{G}+\text{T}\text{G}\right)\times\:\text{B}\text{M}\text{I}}{\text{l}\text{n}\left(\text{H}\text{D}\text{L}\text{-}\text{C}\right)}$$.

METS-IR, employed as a potential mediator in this study, reflects the degree of individual insulin resistance. Consistent with established literature, METS-IR was calculated using fasting plasma glucose (FPG, mg/dL), fasting triglycerides (TG, mg/dL), HDL cholesterol (HDL-C, mg/dL), and BMI (kg/m²). All biochemical measurements were obtained from venous blood samples collected during the NHANES physical examination component and analyzed in certified laboratories following standardized protocols with stringent quality control procedures. Fasting glucose and lipid profiles collected after at least eight hours of fasting were used to ensure the index’s accuracy. Higher METS-IR values indicate more severe insulin resistance. Previous research has demonstrated a strong correlation between METS-IR and insulin sensitivity as measured by the hyperinsulinemic-euglycemic clamp technique, validating its role as a reliable surrogate marker for assessing metabolic insulin resistance.

*Definition and selection of covariates* To mitigate potential confounding, a comprehensive set of covariates was incorporated into the analyses. The selection of these covariates was informed by prior studies and biological plausibility, focusing on factors closely associated with NAFLD and metabolic or obesity-related conditions. Covariates were categorized as follows:

*Demographic and socioeconomic variables* These included age (continuous, in years), sex (male or female), race/ethnicity (categorized according to NHANES classifications as Mexican American, other Hispanic, non-Hispanic White, non-Hispanic Black, or other/multiracial), annual household income^48^ (expressed as the poverty income ratio, PIR, stratified as low-income (PIR < 1.0), middle-income (PIR 1.0–2.9), and high-income (PIR ≥ 3.0)), and educational attainment^49,50^ (less than high school, high school graduate or equivalent, and college or above). These demographic factors are well-established determinants of NAFLD risk, with evidence indicating that advancing age, male sex, racial/ethnic disparities, and lower socioeconomic status may contribute to increased NAFLD prevalence.

*Lifestyle and behavioral variables* Lifestyle factors included alcohol consumption and smoking status. Based on NHANES questionnaire data, alcohol consumption^51^ was classified as never drinkers (lifetime consumption ≤ 12 drinks), former drinkers (no alcohol consumption in the past year but a prior drinking history), and current drinkers (consumption of more than 12 drinks within the past year). Smoking status was categorized as never smokers (lifetime consumption < 100 cigarettes), former smokers (history of ≥ 100 cigarettes but abstinent for at least one year at the time of the survey), and current smokers (actively smoking or abstinent for less than one year)^52^. Both alcohol use and smoking are recognized as critical lifestyle determinants of liver health and metabolic status, and their inclusion accounts for their potential influence on NAFLD risk variation across the population.

*Clinical conditions and anthropometric measures* Several metabolic conditions closely related to NAFLD were accounted for, including categorical variables for hypertension, diabetes, and metabolic syndrome (MetS), along with continuous BMI (kg/m²) measurements to represent overall adiposity. Hypertension was defined as systolic blood pressure (SBP) ≥ 140 mmHg, diastolic blood pressure (DBP) ≥ 90 mmHg, self-reported physician diagnosis, or current use of antihypertensive medications. Diabetes was defined according to standard criteria: fasting plasma glucose ≥ 7.0 mmol/L (126 mg/dL), 2-hour plasma glucose ≥ 11.1 mmol/L (200 mg/dL) during an oral glucose tolerance test, glycated hemoglobin (HbA1c) ≥ 6.5%, or physician-diagnosed diabetes with ongoing treatment. MetS was defined according to the criteria of the American Heart Association and the National Cholesterol Education Program Adult Treatment Panel III (AHA/NCEP-ATP III), requiring central obesity (waist circumference ≥ 94 cm for men and ≥ 80 cm for women) plus at least two of the following five components^53^: (1) elevated blood pressure (SBP ≥ 130 mmHg, DBP ≥ 85 mmHg, or current antihypertensive treatment); (2) hypertriglyceridemia (TG ≥ 150 mg/dL or lipid-lowering therapy); (3) reduced HDL-C (men: < 40 mg/dL, women: < 50 mg/dL); (4) impaired fasting glucose (FPG ≥ 100 mg/dL or diagnosed diabetes); and (5) obesity (central obesity as a prerequisite). These clinical variables were determined through a combination of NHANES questionnaires, physical examinations, and laboratory findings. Their inclusion was essential to account for factors that could simultaneously influence both BFR (exposure) and NAFLD (outcome), thereby reducing potential confounding bias.

*Laboratory biochemical markers* A comprehensive panel of continuous laboratory biomarkers—capturing hepatic and renal function as well as lipid, glucose, and inflammatory profiles—was included to further control for potential confounders. These variables included serum albumin (Alb), alanine aminotransferase (ALT), aspartate aminotransferase (AST), gamma-glutamyl transferase (GGT), total cholesterol (TC), serum triglycerides (TG), HDL-C, fasting plasma glucose (FPG), blood urea nitrogen (BUN), creatinine (Cr), uric acid (UA), total bilirubin (TBil), total protein (TP), globulin (Glob), lactate dehydrogenase (LDH), and hematological indices such as white blood cell count (WBC), lymphocyte count, neutrophil count, red blood cell count, and platelet count. The selection of these biomarkers was based on the pathophysiological characteristics of NAFLD, which is closely linked to liver enzyme abnormalities, insulin resistance, dyslipidemia, and inflammatory responses. All covariates were retained in the multivariable regression models to rigorously adjust for confounding.

### Statistical analysis

All statistical analyses in this study were conducted using R software (version 4.4.0, R Foundation for Statistical Computing). Given the complex sampling design of NHANES, the survey package was used to construct weighted design objects with svydesign, specifying sampling weights, strata, and primary sampling units (PSUs) to account for sampling errors and design effects. For descriptive statistics, continuous variables were presented as mean ± standard deviation or median with interquartile range, depending on distributional characteristics, while categorical variables were reported as frequencies and weighted percentages. Between-group comparisons were performed using weighted independent-sample t-tests, weighted Mann-Whitney U tests, or weighted chi-square tests, as appropriate.To examine the association between BFR and NAFLD, multivariable logistic regression models were constructed with stepwise adjustments for demographic variables, metabolic comorbidities, lifestyle factors, and clinical biochemical indicators. To explore the dose–response relationship between BFR and NAFLD risk, restricted cubic spline (RCS) functions from the **rms** package were applied to model BFR as a nonlinear continuous variable. Segmented regression analyses were performed on the RCS curves to identify the optimal inflection point and to estimate the strength of the association between BFR and NAFLD risk below and above this threshold. To investigate the mediating role of METS-IR, causal mediation analysis was conducted using the “mediation” package, quantifying the indirect effect of BFR on NAFLD through METS-IR. A nonparametric bootstrap method with 5,000 resamples was employed to estimate the average direct effect (ADE), average causal mediation effect (ACME), and their corresponding 95% confidence intervals. The mediation effect was deemed statistically significant when the confidence interval for the indirect effect excluded zero, a widely accepted criterion in mediation analysis. To verify the robustness of these findings, multiple sensitivity analyses were conducted, following best-practice recommendations for assessing the stability of results under alternative assumptions. These included stratified analyses by sex, race/ethnicity, and income level to assess heterogeneity in the association between BFR and NAFLD across subpopulations. Additionally, we tested alternative definitions of NAFLD (e.g., applying different FLI thresholds) and substituted METS-IR with conventional insulin resistance indices such as HOMA-IR. The consistency of these results with our primary analysis supports the robustness and reliability of our conclusions. All statistical tests were two-sided, and P values < 0.05 were considered statistically significant.

## Electronic supplementary material

Below is the link to the electronic supplementary material.

Supplementary Material 1

## Data Availability

Publicly available datasets were analyzed in this study. These data can be found at: https://wwwn.cdc.gov/nchs/nhanes (accessed on 14 April 2025).

## Abbreviations

BFR: Body fat percentage
NAFLD: Non-alcoholic fatty liver disease
METS-IR: Metabolic score for insulin resistance
OR: Odds ratio
CI: Confidence interval
t: t-test
χ²: Chi-square test
BMI: Body Mass Index
WC: Waist circumference
WHR: Waist-hip ratio
T2DM: Type 2 diabetes mellitus
HDL-C: High-density lipoprotein cholesterol
LDL-C: Low-density lipoprotein cholesterol
TG: Triglycerides
TC: Total cholesterol
ALT: Alanine aminotransferase
AST: Aspartate aminotransferase
FPG: Fasting plasma glucose
HbA1c: Hemoglobin A1c
NHANES: National Health and Nutrition Examination Survey
RCS: Restricted cubic spline
DAG: Directed acyclic graph
SE: Standard error
MASLD: Metabolic dysfunction-associated steatotic liver disease
NASH: Non-alcoholic steatohepatitis
GGT: Gamma-glutamyl transferase
BUN: Blood urea nitrogen
Cr: Creatinine
UA: Uric acid
TBIL: Total bilirubin
TP: Total protein
Glob: Globulin
LDH: Lactate dehydrogenase
WBC: White blood cell count
LYMPH: Lymphocyte count
NEUT: Neutrophil count
RBC: Red blood cell count
PLT: Platelet count
PIR: Poverty income ratio
MetS: Metabolic syndrome
FLI: Fatty Liver Index
