# Supplementary material for: Nonlinear relationship between body fat percentage and NAFLD mediated by METS-IR: threshold effects and subgroup differences
Source: Sci Rep. 2025 Jul 10;15:24917. doi: 10.1038/s41598-025-10765-w (PMC12246239; doi:10.1038/s41598-025-10765-w)
Supplement: Supplementary file 1 — Supplementary Material 1 [file 41598_2025_10765_MOESM1_ESM.docx]

Supplementary Table 1. Threshold Effect in the Association Between METS-IR and NAFLD

| Outcome | effect | *P* |
| --- | --- | --- |
|  |  |  |
| Model A Fitting model by standard linear regression | 1.48 (1.45 - 1.52) | <.001 |
| Model B Fitting model by two-piecewise linear regression |  |  |
| Inflection point | 36.066 |  |
| <36.066 | 1.72 (1.23 - 2.42) | 0.002 |
| ≥36.066 | 1.47 (1.43 - 1.51) | <.001 |
| P for likelihood test |  | <.001 |

Supplementary Table 2. Threshold Effect in the Association Between BFR and NAFLD

| Outcome | effect | *P* |
| --- | --- | --- |
|  |  |  |
| Model A Fitting model by standard linear regression | 1.12 (1.11 - 1.13) | <.001 |
| Model B Fitting model by two-piecewise linear regression |  |  |
| Inflection point | 25.103 |  |
| <25.103 | 1.61 (1.43 - 1.83) | <.001 |
| ≥25.103 | 1.09 (1.08 - 1.10) | <.001 |
| P for likelihood test |  | <.001 |

Supplementary Table 3.1 Regression Results: Exposure and Mediator

| Variables | β | S.E | t | P | β (95%CI) |  |
| --- | --- | --- | --- | --- | --- | --- |
|  |  |  |  |  |  |  |
| Intercept | 23.23 | 0.52 | 44.85 | <.001 | 23.23 (22.22 ~ 24.25) |  |
| BFR | 1.35 | 0.03 | 53.80 | <.001 | 1.35 (1.30 ~ 1.40) |  |
| CI: Confidence Interval | | | | | |  |

Supplementary Table 3.2 Regression Results: Exposure and Outcome

| Variables | β | S.E | t | P | OR (95%CI) |  |
| --- | --- | --- | --- | --- | --- | --- |
|  |  |  |  |  |  |  |
| Intercept | -4.62 | 0.20 | -22.89 | <.001 | 0.01 (0.01 ~ 0.01) |  |
| BFR | 0.31 | 0.01 | 28.01 | <.001 | 1.37 (1.34 ~ 1.40) |  |
| OR: Odds Ratio, CI: Confidence Interval | | | | | |  |

Supplementary Table 3.3. Regression Results: Exposure, Mediator, and Outcome

| Variables | β | S.E | t | P | OR (95%CI) |  |
| --- | --- | --- | --- | --- | --- | --- |
|  |  |  |  |  |  |  |
| Intercept | -18.19 | 0.77 | -23.54 | <.001 | 0.00 (0.00 ~ 0.00) |  |
| BFR | 0.10 | 0.02 | 5.28 | <.001 | 1.10 (1.06 ~ 1.14) |  |
| METS_IR | 0.39 | 0.02 | 23.72 | <.001 | 1.48 (1.43 ~ 1.53) |  |
| OR: Odds Ratio, CI: Confidence Interval | | | | | |  |

Supplementary Table 3.4. Mediation Analysis: Indirect, Direct, and Total Effects

| Effect | Estimate | Lower | Upper | β (95%CI) | P | Mediation |  |
| --- | --- | --- | --- | --- | --- | --- | --- |
|  |  |  |  |  |  |  |  |
| Indirect | 0.00 | 0.00 | 0.00 | 0.00 (0.00 ~ 0.00) | <.001 | 84.69 |  |
| Direct | 0.00 | 0.00 | 0.00 | 0.00 (0.00 ~ 0.00) | <.001 | 15.31 |  |
| Total | 0.00 | 0.00 | 0.00 | 0.00 (0.00 ~ 0.00) | <.001 | 100.00 |  |
| CI: 95% Confidence Interval; Lower: Lower limit of 95% CI; Upper: Upper limit of 95% CI | | | | | | |  |

Supplementary Table 3.5. Mediation Analysis: Pathway Analysis

| Pathway | Relationship | β | SE | Lower | Upper | P | β (95%CI) |  |
| --- | --- | --- | --- | --- | --- | --- | --- | --- |
|  |  |  |  |  |  |  |  |  |
| BFR→ METS_IR | Exposure→ Mediator | 1.35 | 0.03 | 1.30 | 1.40 | <.001 | 1.35 (1.30 ~ 1.40) |  |
| BFR → FLI_custom | Exposure→ Outcome | 0.10 | 0.02 | 0.06 | 0.13 | <.001 | 0.10 (0.06 ~ 0.13) |  |
| METS_IR→ FLI | Mediator→ Outcome | 0.39 | 0.02 | 0.36 | 0.42 | <.001 | 0.39 (0.36 ~ 0.42) |  |
| S.E.: Standard Error; CI: 95% Confidence Interval; Lower: Lower limit of 95% CI; Upper: Upper limit of 95% CI Note: Exposure → Outcome represents the direct effect. For total effect, refer to Supplementary Table 3.2. | | | | | | | |  |
